# Energy-Aware Topology Control Strategy for Human-Centric Wireless Sensor Networks

**DOI:** 10.3390/s140202619

**Published:** 2014-02-07

**Authors:** Roc Meseguer, Carlos Molina, Sergio F. Ochoa, Rodrigo Santos

**Affiliations:** 1 Department of Computer Architecture, Universitat Politècnica de Catalunya, Barcelona 08034, Spain; E-Mail: meseguer@ac.upc.edu; 2 Department of Computer Engineering, Universitat Rovira i Virgili, Tarragona 43007, Spain; E-Mail: carlos.molina@urv.net; 3 Computer Science Department, Universidad de Chile, Av. Blanco Encalada 2120, 3er Piso, Santiago 8370459, Chile; 4 Department of Electrical Engineering, Universidad Nacional del Sur – CONICET, Bahia Blanca 8000, Argentina; E-Mail: ierms@criba.edu.ar

**Keywords:** human-centric wireless sensor network, energy consumption, topology control messages, message prediction, participatory sensing, opportunistic sensing

## Abstract

The adoption of mobile and ubiquitous solutions that involve participatory or opportunistic sensing increases every day. This situation has highlighted the relevance of optimizing the energy consumption of these solutions, because their operation depends on the devices' battery lifetimes. This article presents a study that intends to understand how the prediction of topology control messages in human-centric wireless sensor networks can be used to help reduce the energy consumption of the participating devices. In order to do that, five research questions have been defined and a study based on simulations was conducted to answer these questions. The obtained results help identify suitable mobile computing scenarios where the prediction of topology control messages can be used to save energy of the network nodes. These results also allow estimating the percentage of energy saving that can be expected, according to the features of the work scenario and the participants behavior. Designers of mobile collaborative applications that involve participatory or opportunistic sensing, can take advantage of these findings to increase the autonomy of their solutions.

## 1. Introduction

Wireless communication and mobile computing technologies are changing the way in which we perform our everyday activities, and they are already part of our lives. The ‘always connected’ paradigm is more a reality than an expectation. More and more mobile collaborative applications involving opportunistic or participatory sensing are used by people to determine, e.g., the status of the vehicular traffic, the security level of a certain area, and the location of interesting places while performing tourist activities [1–4]. In order to do that, mobile users can utilize their smartphones as a sensor able to detect other computing devices or antennas nearby. Thus, a passer-by could determine the presence and location of friends in the area (e.g., detecting their smartphones) or an interesting museum (e.g., detecting the signal spread by an antenna of the museum). These applications are usually network intensive, therefore their energy consumption becomes a major concern for both users and software designers.

There are two basic approaches used by these applications to perform the sensing activities: opportunistic and participatory. In opportunistic sensing the user is not particularly aware of the activities being performed by his mobile device (e.g., a smartphone) [5], however in a participatory approach the user is an active participant in the sensing activities [1]. In this paper we consider the use of a Human-centric Wireless Sensor Network (HWSN) [6] as the support of the sensing activities, because these networks support both approaches, and also their combinations.

Reducing the energy consumption of the nodes participating in these networks allows the collaborative sensing solutions to increase their availability. This also contributes to improve the trustworthiness of the information provided by them, due a larger number of nodes participate in the sensing process.

In a previous work, the authors proposed to use a predictor of the HWSN topology to help reduce the energy consumption of the nodes participating in these networks [7]. Such a proposal was based on Optimized Link State Routing with prediction (OLSRp) [8] and it included adaptations to be used on a HWSN. The evaluation of such a topology prediction strategy showed promising results in terms of energy savings; however it considered only a particular work scenario. Therefore it is not clear which are the limits of that proposal.

This paper extends that previous work trying to determine how the node mobility, the network degree and the shape of the application area, affect the amount of energy that can be saved when using this prediction strategy. Several simulations were done to determine the impact of these variables. The obtained results help identify favorable scenarios where the topology control (TC) strategy can be used to support these mobile collaborative applications. These results also allow quantifying the energy savings that each participating device can expect, considering the features of the working area and the node behavior.

Designers of mobile collaborative sensing applications can use these results to determine the expected autonomy of the mobile devices, the lifetime of a HWSN, and the trustworthiness of the information provided by these applications. The autonomy and availability of these solutions can be improved using the proposed energy saving strategy. As shown by Ruiz-Lopez *et al.* addressing these non-functional requirements is critical in this type of systems [9,10]. The use of the proposed strategy is not exclusive, therefore it could be used together with other similar proposals to increase even more the energy savings.

The following section briefly describes the main components of a human-centric wireless sensor network and its communication dynamics. Section 3 presents the related work. Section 4 describes the message prediction process that allows HWSN to reduce the energy consumption. Section 5 describes the simulation settings used to determine the limits of this energy saving strategy, as well as the obtained results. Section 6 discusses the findings and identifies the lessons learned. Section 7 presents the conclusions and future work.

## 2. Human-Based Wireless Sensor Network

A HWSN consists of a mesh that allows a variety of sensors to retrieve and disseminate context information in a certain area [6,7]. These sensors can be regular sensors (including smartphones [11]), human-based sensors (e.g., people using a mobile application on a smartphone to detect the presence of friends in the area), mules (e.g., vehicles that—carrying a mobile computing device—connect two or more disconnected networks) and witness units (also known as information sprinklers [12], which can be used to store and deliver information on-demand). However, the main actors in these networks are the human-based sensors, which have the capability of sending/receiving information on-demand to/from other sensors, and also interconnecting the incoming information to produce more complex and accurate knowledge. For example, a shopping center employee (*i.e.*, a human-based sensor) that does not want to take lunch alone nor talk about work during lunch, goes for a walk to find a friend in the neighboring area. He uses the smartphone (*i.e.*, a regular sensor) to detect the presence of a friend's smartphone in the HWSN of the shopping center, which is formed by the mobile devices used by people visiting or working in that place. Thus, the incoming information given by the regular sensor (*i.e.*, the smartphone) is used by the human-based sensor to generate knowledge. Such knowledge is information that can be then shared with other human-based sensors through the HWSN. These sensors types are in fact sensors roles, therefore a certain device can play more than one role in a HWSN. These capabilities of a HWSN can be used to support opportunistic and also participatory sensing [1,5].

These networks are usually composed by several islands of nodes (*i.e.*, sub-networks), which are dynamically created and unified according to the movements of the participants (*i.e.*, the network nodes). On the one hand, if we consider each island as a single node, then the HWSN behaves as an opportunistic network (oppnet). This means that the nodes are able to route messages from a source to a destination node, without knowing if there is a path between them [13]. On the other hand, each island behaves as a mobile *ad hoc* network (MANET), where the existence of path from the source to the destination node is previously known. This behavior determines that the network topology is never known by the nodes, unless that all of them belong to a single island.

Message routing among islands typically requires the participation of mules, which implement store and forward mechanisms. However, this propagation activity can also be performed by regular nodes. Several criteria can be used to choose the nodes that will participate in this activity; for instance, those with the highest mobility level [14–16] or probability of meeting the destination node [17], those with similar profiles [18], or any other criterion. These nodes add the capacity of storing (in memory) the messages that cannot be delivered, because the destination node is outside the island. These messages are kept by the nodes until a suitable “carrier” is found. The carrier forwards them to other islands. The propagation process is done until the destination node is met, or *the time to live* of that message is reached.

The topology control (TC) strategies used in HWSN-based applications usually plays an important role in both the message dissemination and energy consumption inside the islands. Designers of these applications must decide what is the best way to not only keep the sub-network topology updated in this dynamic scenario, but also to reduce energy consumption of the participating devices. The chosen strategies should not interfere with the regular traffic of the network. In general, these networks are built upon an IEEE 802.11 variant [19]. The IP assignment for the network nodes is outside the scope of this paper, but it can be performed following the proposal described in [20].

The study presented in this paper considers the use of a MANET for the message delivery inside the islands of a HWSN. The messages in the network islands are routed using OLSRp [8], which considers the use of a TC message predictor as the main strategy to reduce the energy consumption of the nodes. These nodes typically keep a local routing table that allows them to determine if a certain node is or not in the island. Mobile collaborative applications can (indirectly) ask the local routing table to determine if a certain mobile node (e.g., a friend of the shopping center employee) is in the island, and thus determine appropriate periods to send him messages. Clearly if the application decides to deliver a message when the destination node is not reachable, such a message will consume energy and computing time of the interim nodes without a purpose until *the time to live* is reached. Therefore, mobile collaborative applications can reduce their energy consumption not only because the use of a TC message predictor, but also due the TC information can be used at the application level to identify appropriate periods to deliver messages to other mobile users.

## 3. Related Work

Several strategies have been proposed to reduce energy consumption in WSNs, however only some of them are suitable to be used in a HWSN, as they take into account the node mobility and weak communication links among them. Mahfoudh and Minet present an interesting survey of energy efficient strategies to maintain the topology of mobile *ad hoc* and sensor networks [21]. Several of these proposals could be eventually used to reduce energy consumption in the islands of a HWSN. These energy saving strategies can be classified in four categories: *energy efficient routing, topology control by tuning the node transmission power, scheduling the nodes sleeping state*, and *reducing the volume of transferred information*. Concerning the first category, Tekbiyik and Uysal-Biyikoglu [22] present a survey of energy-efficient routing strategies for machine-to-machine networks. Some of them could also be used in HWSN. Maleki *et al.* [23] propose the Lifetime Prediction Routing protocol for mobile ad hoc networks, where each node tries to estimate its battery lifetime on the basis of its past activity. Hence, it is possible to increase the overall network lifetime by finding better routing solutions that take into account these predictions. Several other proposals have been reported in this category, for example [24–26]. These proposals either explore routes that include nodes with maximum residual energy, minimize the total end-to-end transmission energy for a message, or a (weighted) combination of both strategies.

Most energy efficient routing strategies are not exclusive, therefore they could be combined properly with OLSRp to increase the energy saved by the network nodes. However, it is not clear what effect those combinations can have on the network traffic, particularly on the data throughput and packet delivery ratio.

The Kinetic Multipoint Relaying protocol [27] focuses on predicting the node mobility to improve routing and save energy. This approach selects relay nodes on the basis of the current relay configuration and the future network topology prediction. Similar to the previous category, the proposals that use tuning of the nodes transmission power can also be combined with proposals that reduce energy consumption using a predictor.

Concerning the strategies based on the use of sleeping nodes, Ye *et al.* [28] proposed a robust energy-conserving protocol applied to a very large number of small sensors with short battery lifetime. The protocol deals with geometric information to derive redundancy, and thus it allows redundant nodes to be turned off. A similar strategy has been used by Tian and Georganas [29], by Chen *et al.* [30], and by Xu *et al.* [31] in wireless *ad hoc* networks. The proposals in this category do not make sense for use in HWSN, due to the fact that all nodes are important actors in the sensing process. Therefore, reducing the number of available nodes will only jeopardize the interaction among devices, and decrease the trustworthiness of the information obtained from the sensing process.

An interesting strategy that reduces the volume of transferred information was presented by Le *et al.* [32]. They propose a new MAC technique for event-driven wireless sensor network, which creates a hierarchical topology where every node synchronizes the information with its neighbors to avoid resending the same data. In this way, if a node receives the same information that was sent to the same destination, it will remove this message as it assumes that it has already been sent by one of its neighbors, and thus the nodes save energy.

Hong *et al.* [33] and also Iwata *et al.* [34] have made particular proposals to reduce the information overhead in MANETs, thus, dealing with the problem of energy consumption in mobile devices participating in oppnets. However, none of these proposals use message prediction as the instrument that allows reaching such a goal, which is the particular strategy analyzed in this study.

There are also other proposals that do not fit into the previous categories. For example, De Rosa *et al.* [35] propose the Mobile Gambler's Ruin algorithm that uses node mobility prediction to save energy. This predictive algorithm assumes a cooperative scenario, in which it tries to identify nodes that will more likely be disconnected in the near future. Therefore, this prediction allows the coordination layer to re-schedule the work among nodes in advance. However, using this algorithm does not ensure that nodes will have an updated version of the network topology when they need to send a message. This would impact negatively on the network throughput.

Montolio-Aranda *et al.* [36] propose a strategy to reduce resources consumption in wireless ad hoc networks when broadcast messages are used to establish network topologies and routing tables. The proposal is based on two key concepts to perform the message flooding: the distance-enabled multipoint relaying and the connected dominating set structure. The main limitation of this proposal is the flooding of broadcast messages, which is also a resource consuming process. Cervera *et al.* [37] and also Abdou *et al.* [38] improve the flooding strategy in this scenario, trying to enhance the message delivery more than reducing the energy consumption of that process. Rodriguez-Covilli *et al.* present a promising topology control strategy, which is particularly appropriate for MANETs involving nodes with high mobility [39]. Unfortunately such a proposal does not optimize the energy consumption of the topology control process.

In [40], the authors try to improve the reliability and availability of WSNs by proposing a data forwarding algorithm. The approach is useful when the traffic network is not high. The proposal does not consider human-based sensors or mobility of the nodes. In [41], the authors introduce the concept of quality of service (QoS) for the routing analysis in WSN and how it impacts in the energy consumption, delay and latency in the network. The analysis of QoS performance is not directly included in that paper, but it is shown how the utilization of the proposed algorithm extends the life of the sensors. This obviously has a positive impact on the network performance.

Many proposals have been done to implement routing in oppnets, but most of them are not concerned of the energy consumption involved in the topology control or message dissemination process. Provided these networks do not ensure the existence of a known path between the source and destination nodes, most proposals implement routing strategies based on the store and forward paradigm [14–18]. In all cases, these routing strategies try to use information about the network topology and node behavior, in order to determine which nodes will act as gateways (*i.e.*, nodes performing the store and forward of the messages). Typically, the network nodes keep a record of the visited places, announced profiles (e.g., likes and dislikes), mobility information (e.g., direction and speed), or encounters with other nodes. This information is used to determine the best nodes to perform the store and forward the messages. The idea is to maximize the probability of meeting the destination node. By doing so, it is also expected to reduce the energy used during the transmission process or the number of message copies in the network.

Most proposals consider the oppnet built from a set of disconnected meshes or local communities (*i.e.*, islands). Messages within the community are routed following some traditional routing technique, for which the topology of the local sub-network is needed. For instance, the Context-Aware Adaptive Routing [16] uses *dynamic destination-sequenced distance-vector* [42] for doing the local routing. In this paper, we analyze the impact of using OLSRp on HWSNs, which are implemented combining oppnets and MANETs. The energy saving strategy used in OLSRp is based on the prediction of TC messages, and it is briefly described in the next section.

## 4. OLSR with Prediction

The Optimized Link State Routing (OLSR) protocol [43] is a well-known proactive routing protocol for *ad hoc* networks. The nodes in these networks periodically exchange routing information to maintain a map of the network topology. The Multi-Point Relays (MPRs) are the network nodes selected for propagating the topology information. In OLSR, there are two types of control messages: HELLO and Topology Control (TC). HELLO messages allow each node to discover its neighboring nodes and obtain information about the state of the links with them. TC messages allow MPR nodes to disseminate neighbor information throughout the network. The mathematical modeling that helps understand the MPR selection and topology dissemination (using TC messages) processes is described in [44]. In [45] the authors provide a mathematical description of the neighbor nodes discovery and link failure detection (using HELLO messages) processes.

Topology control message prediction can be performed in different ways, and it does not necessarily need to rely on the past history. However, a repetitive behavior of the nodes facilitates the prediction process. The simplest approach is to use, as a predictor, the “last-value” observed by a node. This takes advantage of the values repetition, which in certain work scenarios can represent quite well the real behavior of the network nodes.

Medina *et al.* [8] have identified that this repetition (*i.e.*, last received message is equal to the preceding one) in OLSR topology control (TC) messages is independent of the number of network nodes, but it is affected by the node mobility, speed and density. Based on these observations, the OLSR routing algorithm was adapted to predict (when possible) the TC messages that nodes need to exchange for building an accurate map of its sub-network topology in HWSNs. Thus, the nodes can avoid resending the same information, which has two important advantages: (1) a reduction of energy consumption and CPU processing time, because fewer routing TC messages are sent and received; and (2) a reduction of the network collisions, because only non-redundant routing TC information is delivered, thus reducing the message traffic.

**Figure 1.**
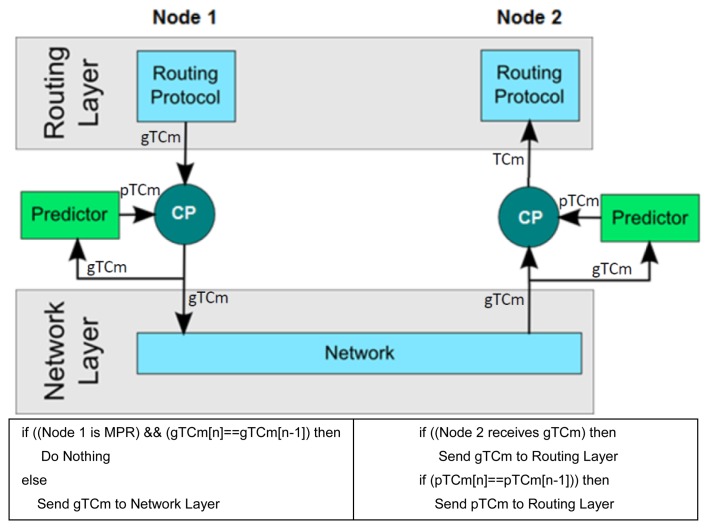
Network architecture including the predictor.

The message prediction process involves the use of a *predictor* component, which is located between network and routing layers. Figure 1 shows the interlayer communication of a node that is implementing the OLSRp system. The OLSRp can be implemented as a transparent communication intermediary between routing and the network layers. Notice that both approaches (*i.e.*, OLSR and OLSRp) deal with exactly the same control traffic. The main difference is that the data sources for the OLSR layer are different. When the OLSR is used alone, all the information comes from the network layer, whereas when the OLSRp is used, the information can be provided by both the network layer and the predictor component.

In order to exemplify the way in which OLSRp works, let us consider the relationship between two nodes belonging to the same island of a HWSN. Typically, a “Node 1” periodically generates a TC message that we will name *generated TCm (gTCm)*. Following the same pace, the predictor in such a node predicts the TC message, based on the last value generated from this node. We will name these messages *predicted TCm (pTCm)*.

The system will deliver a new TC message to other network nodes (including Node 2) as a result of applying a merge algorithm that considers the generated and predicted messages. Moreover, this occurs if both messages (*i.e.*, *gTCm* and *pTCm*) do not contain exactly the same control information. After any delivery, the predictor stores locally the new TC message, which will be used for next predictions.

The Node 2 periodically receives TC messages from other nodes through the network layer. These messages are also considered as gTCm by the Node 2. At the same pace the predictor of that node determines the predicted TCm, *i.e.*, the next TC message that should be received from other nodes (in this case, from Node 1). If no gTCm message is received through the network from a certain node, the routing protocol processes the pTCm. In other case, the routing protocol processes the received gTCm message, and the predictor keeps a local copy of it for the next predictions.

Mobile collaborative systems can take advantage of this TC message prediction to increase their energy savings. Typically these systems exchange messages, at the application level, between nodes that can be in the same island or in others. The ideal situation from an energy consumption point of view is that the sender delivers the message when the destination node is reachable. This situation is detectable if the sender receives a gTCm from the destination node. In other case, the sender can delay the message delivery, as much as possible, waiting for a gTCm. If a gTCm is not received after the waiting period, the sender will have to decide if the message will be sent. Depending on the waiting period and node mobility, the sender could assume that the lack of a gTCm indicates that the target node has left the island, and therefore the sender could discard the message to save energy.

In a previous work the authors showed that the TC message prediction strategy helps reduce the energy consumption of the nodes in a HWSN [7]. However, these results were obtained considering a particular setting, therefore it is not clear what are the limits of this proposal. In order to try identifying the potential impact of this energy saving strategy on HWSN we have defined five research questions:

**RQ1:** How does the node density affect the energy consumption?
**RQ2:** How does the speed of network nodes affect their energy consumption during the topology control process?
**RQ3:** How does the mobility pattern of the nodes affect the energy consumption?
**RQ4:** How does the network degree influence the energy consumption of the nodes?
**RQ5:** How does the shape of the area where the nodes are deployed influence the energy consumption of the nodes?

Several simulations have been done in this study trying to answer these questions. The following section describes the simulation settings and presents the obtained results.

## 5. Simulation Scenarios

The ns-2 [46] simulator was used during this validation process because it allows us to model several network scenarios and collect statistics through trace files. The OLSR model and also the model used by the ns-2 simulator to evaluate the network performance are described in [47].

Using this simulation tool we defined several network topologies, configure wireless network interfaces and set the node mobility patterns. The node mobility was modeled using the BonnMotion simulator [48], due this tool includes well-known models that represent people mobility in the studied scenarios. The mobility models considered in this work have a widely analyzed and validated mathematical basis [49–51].

**Figure 2.**
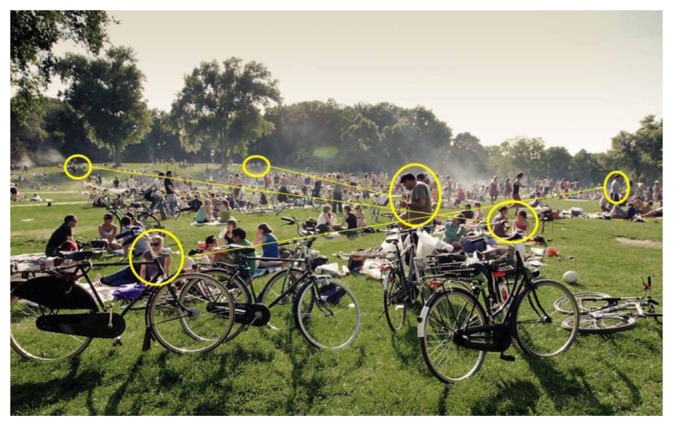
Potential interactions scenario in a park.

For the simulations, we have considered two physical areas that represent regular interaction spaces in our everyday life. The first one is a square open area of 300 × 300 m that represents a beach area or a park (Figure 2), where people are free to move throughout the whole space, and eventually interact with other people (e.g., friends or relatives) in that area. In this scenario the people (*i.e.*, the nodes of a HWSN) can remain stationary during a picnic, or walking with or without a clear direction. Figure 2 shows some matches between people that know each other (*i.e.*, human-based sensors). A mobile collaborative application that detects the presence of these people can eventually trigger impromptu interactions among them.

**Figure 3.**
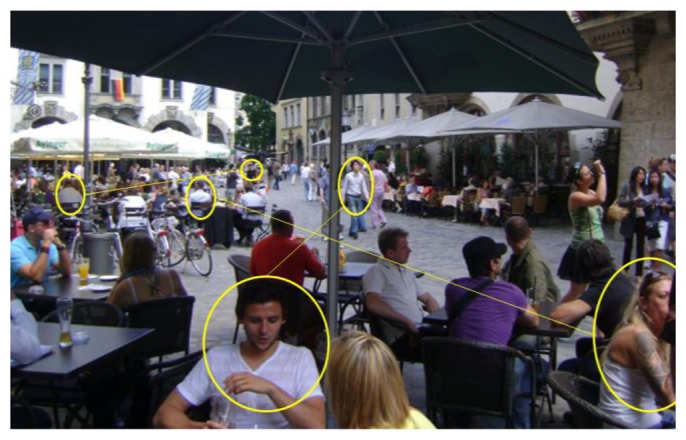
Potential interaction scenario in a pedestrian promenade.

The second scenario is also an open area, similar to a pedestrian promenade, but represented by a rectangle of 30 × 3,000 m. In such a scenario the people can stay for a while (e.g., sharing a drink) or walking through the area (Figure 3). In this place, most pedestrian movements follow a pathway, and people can move in both directions. Both interaction scenarios have the same size (90,000 square meters), which allows us study the influence of the area shape on energy consumption, by keeping the same node density in the interaction space.

The simulations in those scenarios considered a HWSN composed of 10, 20, 30 and 40 nodes respectively, which form various islands according to the people movements. The behavior of these nodes alternate between some periods where they are stationary and others in which they move up to 1, 2, 4 or 6 m per second (mps). The nodes movement can follow one of the following mobility models: *Random Walk*, *Self-similar Least Action Walk* (SLAW), *Nomadic* or *Reference Point Group Mobility* (RPGM) [48]. These mobility models are quite representative of the movements that we can expect of a person or those of a group in the studied scenarios.

The Random Walk model considers people moving randomly in terms of direction and speed, within a certain area [49]. This model illustrates the movements of people in a park, where each person can move via walking, running or riding a bicycle without using formal paths.

In the SLAW model the people move quite randomly, but they are aware of the speed and direction of their movements. This is similar to the movements of people that use the walking paths of a park. This model is also effective in representing casual encounters among community members; e.g., students at the university campus or friends in a theme park [50].

The Nomadic model considers people moving in groups from one location to another. This is representative of guided tours at a city downtown or a museum, where the tourists move together visiting several interest points [49]. This model considers a particular node per group (*i.e.*, a reference node) that determines the next target point, and also a reference path and speed to reach such a place. Such a role can be played by the tour guide.

The RPGM model is similar to the Nomadic, however in this model there is no reference node; therefore the entire group determines its motion behavior (including location, speed, direction, acceleration, *etc*.) according to its goals [51]. This model can be used to represent the movements of search-and-rescue teams during disaster relief efforts, where different teams perform the same operations (*i.e.*, search and rescue survivors) in different sectors of the affected area [49].

In this study we considered a HWSN based on WiFi (IEEE 802.11 standards). Fifteen simulations were done for each scenario and nine of the most representative simulations where used in this study in order to avoid the influence of the special cases. The results shown in this section are the average values of these nine selected simulations, thus we have tried to normalize the network behavior. All simulations were done using OLSR (the standard protocol), and then repeated using OLSRp (the protocol with the predictor).

We have assumed that all nodes are similar, and with technical features equivalent to a smartphone. Particularly, we have considered the features of the iPhone 4 for stationary and mobile nodes. These devices have an effective WiFi communication range of 80 m approximately in open areas. In such range we can expect that the ad hoc communication among devices is stable and the bandwidth of at least 50 Kbytes, which is appropriate to support reliable synchronous interactions among mobile nodes.

The routing protocols were set to deliver HELLO messages every two seconds and TC messages every three seconds. We also generated data traffic (*i.e.*, messages with content generated by the mobile application) that consists of several UDP messages transmitted every second. Table 1 summarizes the simulation parameters, and Table 2 indicates the parameters used for each node's mobility model. The next sections present the results obtained in the square and the narrow interaction area; *i.e.*, interaction scenarios that are representative of a park and a promenade respectively.

### 5.1. Case Study 1: Interactions in a Square Area

As mentioned before, the simulations in this case study considered four alternatives for the node mobility. A first analysis of the obtained results allowed us identify that the energy consumption in the HWSN follows the same pattern when people move according to a Random Walk or a SLAW model. The same situation occurs when people's movements adhere to a Nomadic or a RPGM mobility model. For that reason, the following sections present the simulation results taking into account these two groups.

#### 5.1.1. Simulations Using Random Walk/SLAW

**Figure 4.**
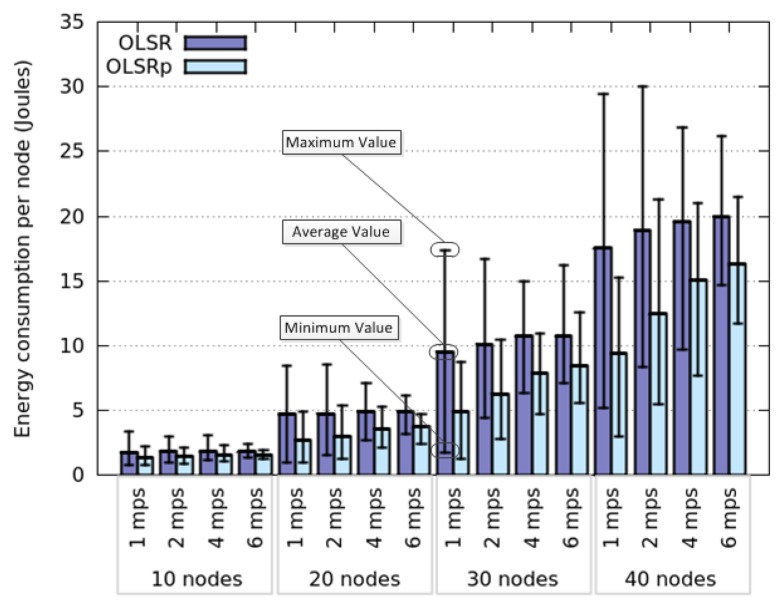
Energy consumption when nodes follow a Random Walk model.

Figure 4 represents the average energy consumption by node, using OLSR and OLSRp, when these nodes move according to a Random Walk model. These simulations consider several densities of nodes (*i.e.*, 10, 20, 30 or 40 nodes in the area) and also node speeds (*i.e.*, 1, 2, 4 and 6 m per second). The results obtained for HWSN deployed in this open area, indicate that the use of the predictor helps reduce energy consumption by 31% (average).

Considering the average energy consumption we see four groups classified according to the number of nodes participating in the network. Clearly this consumption increases with the number of nodes in the area, which provides an initial insight about a possible answer to the RQ1 (*How does the node density affect the energy consumption?*). This is an expected result, since increasing the node density will increase the number of links among nodes; therefore each participant will have to route an extra traffic that impacts on the energy consumption. Although this behavior is present when using both protocols (*i.e.*, OLSR and OLSRp), the participation of the predictor helps reduce the slope of the energy consumption. This means that OLSRp improves the performance of the HWSN (from an energy consumption point of view) in this scenario, and such improvement increases with the node density.

The node speed in this open area seems to have negligible impact on the average energy consumption when OLSR is used. However, its impact is more interesting when using OLSRp. Moreover, the dispersion of the energy consumption decreases when the node speed increases. These results provide a preliminary answer to the RQ2 (*How does the speed of network nodes affect their energy consumption?*).

Considering the maximum and minimum values of energy consumption we can see that the predictor tends to reduce the peaks, probably saving TC messages of nodes that are highly connected (e.g., central nodes of the HWSN) and that involve low mobility. Moreover, the percentage of this reduction seems to be directly related to the dispersion level of the energy consumption in each scenario. The larger the energy consumption dispersion, the larger the energy savings produced by the predictor.

**Figure 5.**
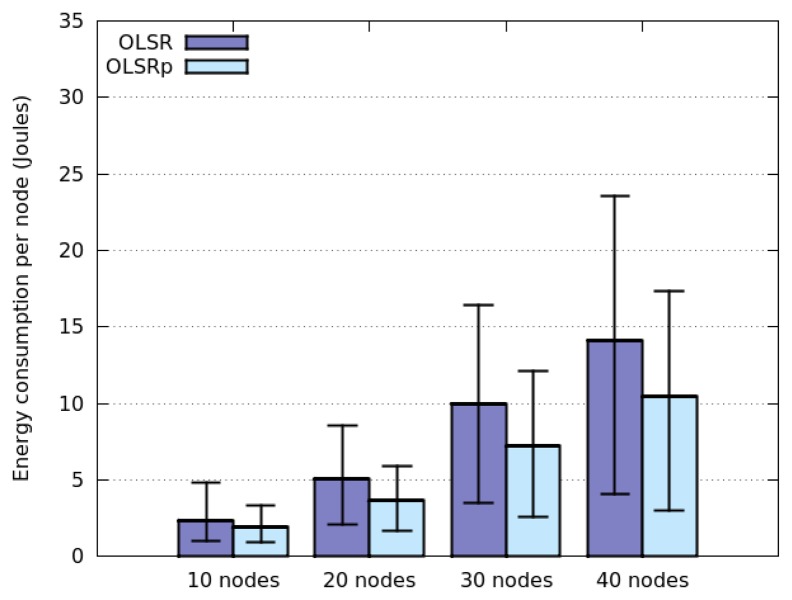
Energy consumption when nodes follow a SLAW model.

Similar results are shown in the simulations when the nodes use a SLAW mobility pattern (Figure 5). In this case the node speed is not a variable of the motion model, due to the nodes moving according to their recent story [52]. Therefore, they do not abruptly change their direction or speed. For that reason their energy consumption is considerably less than in the Random Walk model. Nodes in SLAW tend to move together and keep the network topology quite stable, thus they save TC messages (and energy). It provides an initial answer to the RQ3 (*How does the mobility pattern of the nodes affect the energy consumption?*).

**Figure 6.**
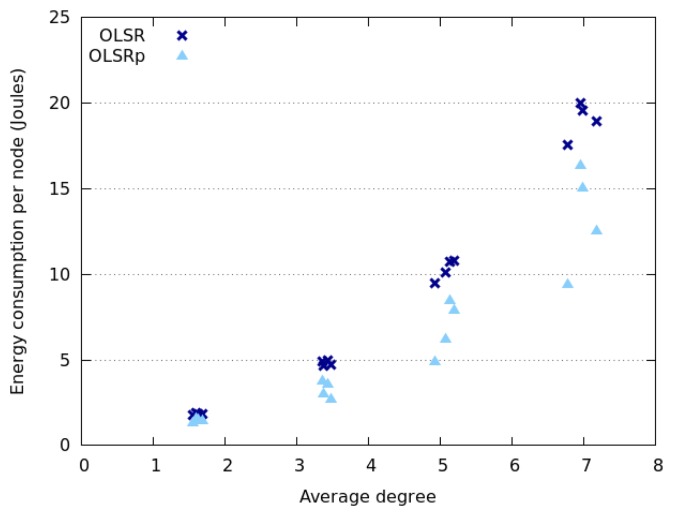
Energy consumption *versus* network degree when nodes follow a Random Walk model.

**Figure 7.**
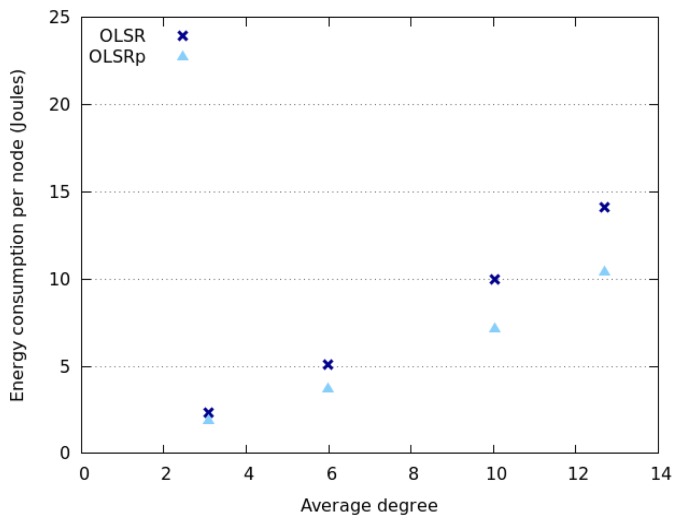
Energy consumption *versus* network degree when nodes follow a SLAW model.

Figures 6 and 7 indicate how the network degree affects the average energy consumption of the nodes in the previously presented interaction scenarios. This uncertainty was specified as RQ4. The results show that the network degree seems to be related to the number of nodes deployed in the area, which is not surprising due to the fact that we are considering a square area. Moreover, energy consumption behaves slightly different when considering Random Walk model and SLAW model. On the one hand, the average energy consumption increases linearly with the network degree when nodes move following a SLAW model. On the other hand, the average consumption grows exponentially when nodes assume a Random Walk model.

The nodes that follow a SLAW model tend to have a network degree higher than those adhering to a Random Walk mobility model. This can be explained because in SLAW the nodes tend to make small changes in their direction and speed, therefore they do not use the whole available space (*i.e.*, they are less scattered in the physical scenario) [52]. On the contrary, when nodes use a Random Walk model, the network tends to be larger and have less links. This situation also explains why nodes moving according to SLAW consume less energy than those adhering to a Random Walk model. In any case, it seems that OLSRp increases the energy savings when increases the number of nodes in the network islands.

#### 5.1.2. Simulations Using Nomadic/RPGM

**Figure 8.**
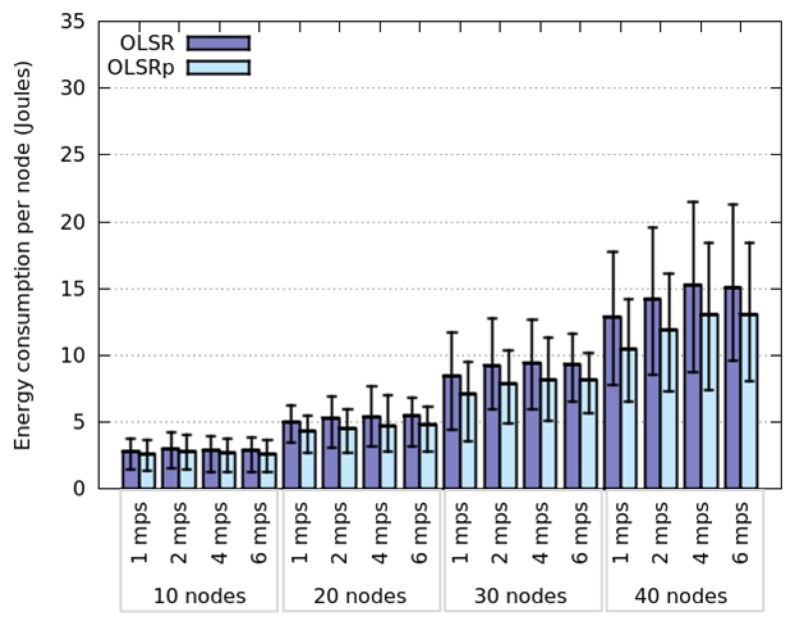
Energy consumption when nodes follow a Nomadic model.

**Figure 9.**
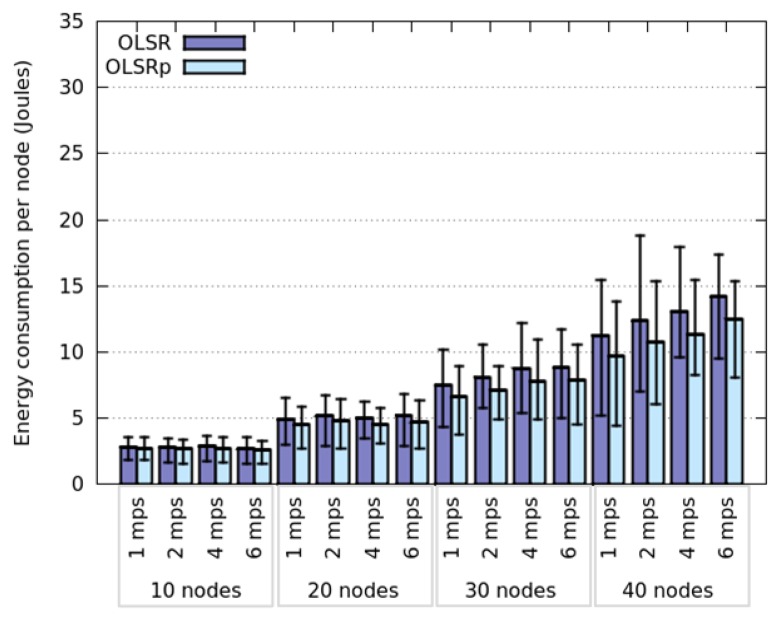
Energy consumption when nodes follow a RPGM model.

This section presents the same simulation scenarios than the previous one, but involving nodes that move following a Nomadic or a RPGM model. Figures 8 and 9 show the obtained results for energy consumption considering these mobility patterns. Compared to Random Walk and SLAW models, the Nomadic and RPGM models show a significant global reduction of the energy consumption, particularly when number of nodes increases. This can be explained because latter models determine its behavior based on groups. This means that nodes inside a group are close enough to be one hop away among them, minimizing thus the number of TC messages and the energy consumption. The simulation results obtained using Nomadic and RPGM supports the observations made on the relationship between node speed and energy consumption dispersion. Moreover, the amount of energy saved by the predictor is not directly related to such dispersion. In this case the predictor seems to show a consistent energy reduction rate of approximately 13%.

**Figure 10.**
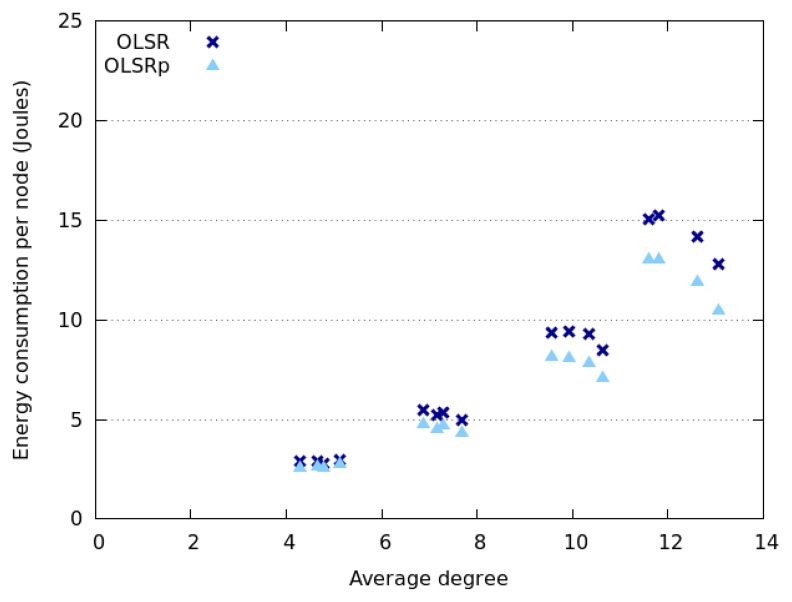
Energy consumption *versus* network degree when nodes follow a Nomadic model.

**Figure 11.**
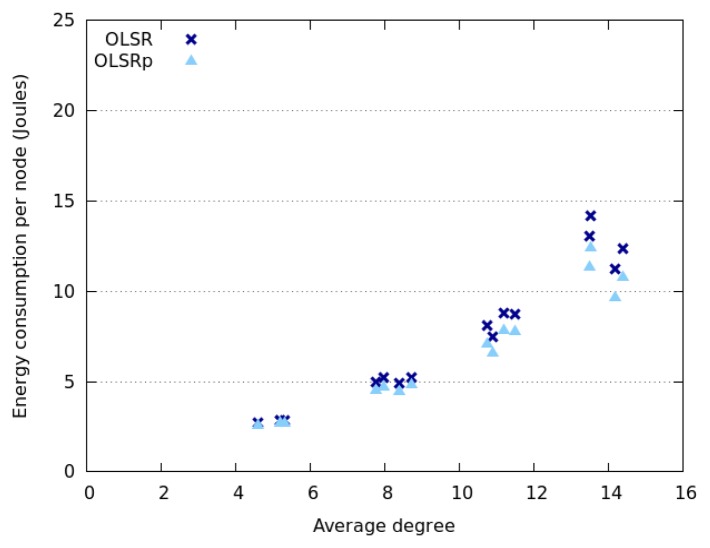
Energy consumption *versus* network degree when nodes follow a RPGM model.

The average energy consumption of the nodes and its dispersion is similar for both mobility models, particularly when the network has a low density of nodes (*i.e.*, 10 or 20 nodes in the area). However when the network overcomes a certain network degree (that number seems to be 8), the RPGM model tends to consume less energy and have a dispersion less than in the Nomadic model. This situation can be observed clearly comparing Figures 10 and 11. Provided that the differences in their performance are so small, we could consider these two motion models as similar (in terms of energy consumption) for this scenario. The next section presents and discusses the simulation results obtained in the narrow working area.

### 5.2. Case Study 2: Interactions in a Narrow Area

In this section we present the results of the simulations done using the same settings as in the previous section, but in this case the nodes were deployed in an area 30 × 3,000 m. The main goal to repeat the simulations was to try understanding the influence that the shape of the working area has on both, the node energy consumption and the energy saving produced by the predictor. Next we present the obtained results considering the studied mobility patterns.

#### 5.2.1. Simulations Using Random Walk/SLAW

**Figure 12.**
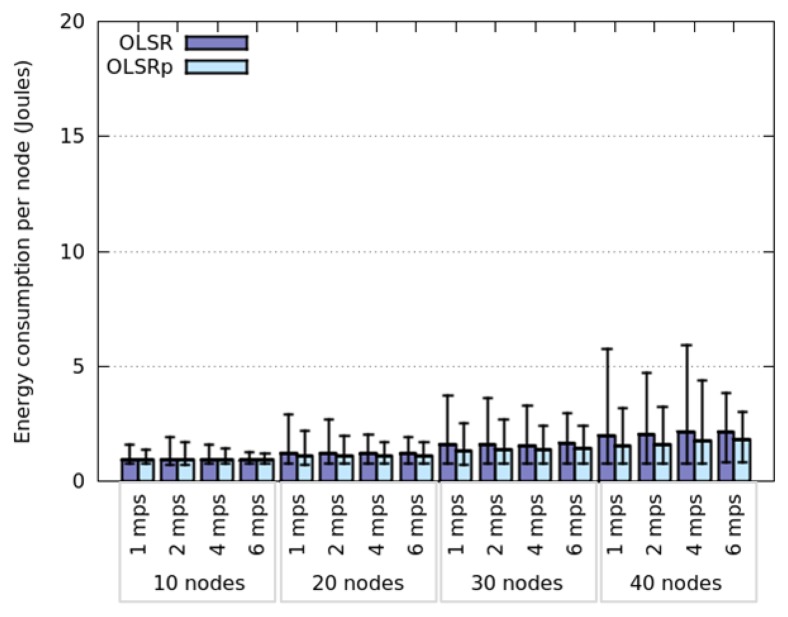
Energy consumption when nodes follow a Random Walk model.

In this scenario, nodes adhering to a Random Walk or SLAW model have shown an almost identical energy consumption and network behavior. This happens because the working area is too long and therefore the node encounter rate is really low. This means that most of the time the nodes are isolated, therefore they do not have to disseminate TC messages and thus they save energy. Evidence of this situation is shown in Figure 12, where we can see that the nodes have low energy consumption, regardless of the network node density.

**Figure 13.**
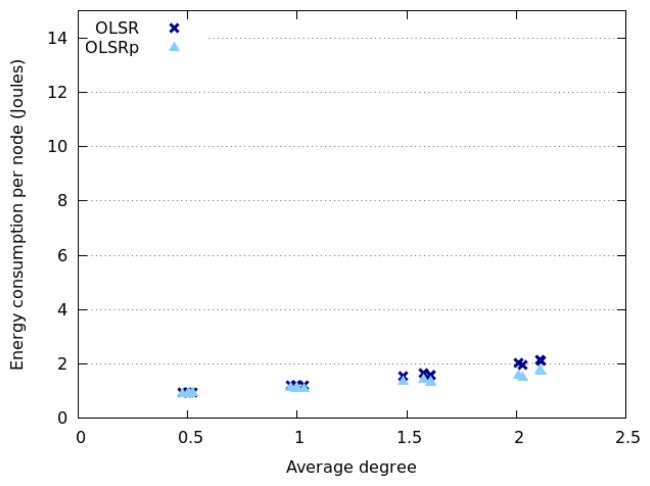
Energy consumption *versus* network degree when nodes follow a Random Walk model.

The network degree shown in Figure 13 also supports this finding. Therefore we can say that the shape of the working area definitively affects the node encounter rate, and the node energy consumption (RQ5). This consumption and the benefits of using the predictor tend to increase with the network degree. Contrary to the results obtained in the square area, the consumption dispersion does not depend on the node speed and the energy saved is not related to the dispersion of the node energy consumption.

The simulations in this scenario allow us to realize that the key aspect to be considered when analyzing the energy consumption (or the node meeting rate) is the network degree instead of the node density. In narrow areas, a higher number of nodes will be close to a border, therefore part of their communication threshold will be outside the interaction area. This means that the potential influence of these nodes would be different depending on where they are located. Therefore, the metric to be considered when determining node meeting rates and energy consumption (during the TC process) should be the *network degree* or the *density of node communication areas*. Each of these areas is defined as the physical space covered by the communication threshold of a node.

In order to determine how narrow a working area is, it is important to consider the relationship between the node communication threshold and the shortest distance between the borders of the considered area. In case of irregular areas, they must be split in regular sub-areas to analyze the node energy consumption.

#### 5.2.2. Simulations Using Nomadic/RPGM

**Figure 14.**
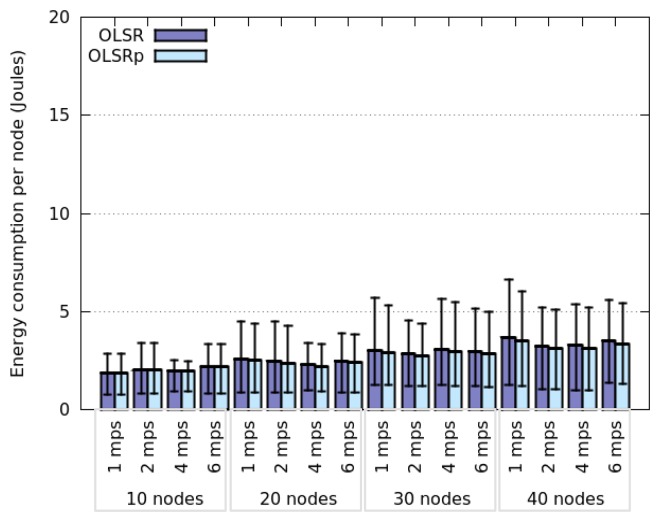
Energy consumption when nodes follow a RPGM model.

**Figure 15.**
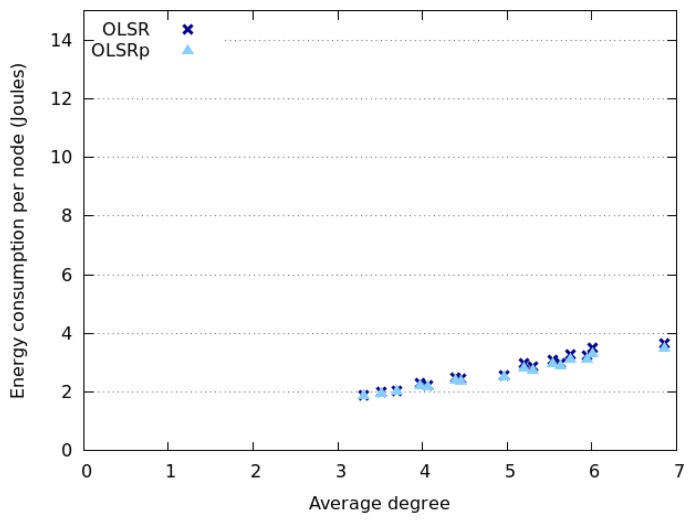
Energy consumption *versus* network degree when nodes follow a RPGM model.

The results obtained in the scenario of narrow shape, when the nodes move according to a Nomadic or a RPGM model, are almost identical. Here we can also see that the groups were isolated most of the time and they had some sporadic encounters with each other. Figures 14 and 15 present evidence of this. Considering that each group has (in average) four members, the network degree indicates that in the best case the network was split in several sub-networks composed just of two groups.

The reduction of the energy consumption in this scenario is really low, because there are almost no topology changes. This happens due to group members are moving together with almost no encounters between groups. Therefore, few TC messages are disseminated, and the dissemination process involves few nodes due to the network is split. In the next section we summarize the obtained results and present the preliminary answers to the stated research questions.

## 6. Discussion

Analyzing the simulation results we can say that the predictor helps reduce the energy consumption of the nodes during the topology control process in all studied scenarios. In the square area the average reductions of the energy consumptions were the following: 30.1% when using Random Walk, 26.6% for SLAW, 14.7% for Nomadic 11.7% for RPGM. Clearly, when the nodes follow a Random Walk model, their motion behavior is independent of other nodes. Therefore the network becomes larger and a higher number of TC messages are disseminated. The energy consumption and its dispersion increases, and also increases the energy saved by the predictor.

The SLAW model represents an interim point between a random and a group mobility model, because the nodes move randomly (in terms of speed and direction) taking into account their recent history. Therefore, they finally tend to move coordinately with some other network nodes, which reduce the number of changes in the network topology and also the energy consumption due to the dissemination of TC messages. For that reason the energy saved by the predictor in SLAW is less than in the Random Walk model. Following the same reasoning we can understand why in group motion models (*i.e.*, in Nomadic and RPGM) the predictor can reduce between 11% and 15% of the energy consumed by a node during the topology control process. Here, each group behaves as a single large node (or network island) that moves randomly through the working area, producing sporadic encounters between groups. This situation becomes evident, observing the network degree in the previously presented scenarios.

On the other hand, the reduction of the energy consumption produced by the predictor was low in the narrow area, as most nodes remain isolated due the shape of the simulated place. In the simulations that used the Random Walk and SLAW, the nodes become isolated, and in Nomadic and RPGM, the groups are isolated. The average reductions in this scenario were the following: 15.3% when using Random Walk, 12.6% for SLAW, 4.7% for Nomadic, and 3.6% for RPGM. The influence of the predictor is clearly different depending on the features of the interaction scenarios. In order to understand the limits of the TC message prediction strategy, we next present the preliminary answers to the stated research questions.

Concerning the *RQ1* (*How does the node density affect the energy consumption?*), we can say that the density is not an appropriate metric to understanding the energy consumption, due to reasons previously mentioned. In that sense the network degree or the density of the node communication areas are better predictors of this energy consumption. Evidently, the energy consumption per node increases with the network degree; however, the way in which it rises depends on other variables; e.g., the motion behavior of the nodes or the shape of the working area. More connected mobile nodes tend to produce more topology changes and therefore the predictor is able to save a higher amount of energy. This also answers the *RQ4* (*How does the network degree influence the energy consumption of the nodes?*). This network degree seems to be independent of the node speed, and dependent on the density of the node communication areas.

Concerning the *RQ2* (*How does the speed of network nodes affect their energy consumption during the topology control process?*) the nodes adhering to a group motion model (*i.e.*, Nomadic or RPGM) tend to increase their energy consumption with the speed, but just in scenarios where they have frequent encounters with other groups. This tendency is stronger in motion models where the nodes move more independently from other nodes (e.g., in the Random Walk model). If the nodes move slowly, the network topology will have few changes and therefore it becomes more predictable. In these cases, the use of the predictor makes an important difference in the energy consumption of the nodes.

Concerning the *RQ3* (*How does the mobility pattern of the nodes affect the energy consumption?*) the first aspect that we have to remark on is that the group motion models are clearly different from those where the nodes move in an independent way. As mentioned before, we can model these nodes groups as large nodes (*i.e.*, islands) that totalize the energy of the group members, and that move randomly through the working area. By making these considerations, the group motion models behave similar to the Random Walk model in terms of node energy consumption. Clearly the energy consumption during the topology control process reduces if we increase the density of network nodes.

Concerning the *RQ5* (*How does the shape of the area where the nodes are deployed influence the energy consumption of the nodes?*) we can say that the shape of the working area is important, because it contributes directly to determining the node encounter rate. The number of TC messages delivered through the network (and the node energy consumption) will depend on such a rate. Therefore, a higher rate would allow the predictor to save a higher amount of energy.

The experiments reported in this article are representative of people moving according to the described models in open areas. The experimentation settings do not consider signal interference or temporal obstacles that could be present in urban areas; therefore the simulation results should be considered as set of valid tendencies instead of absolute values. The experimentation in real scenarios would allow us to understand the magnitude of the difference between the real and the simulated scenarios.

## 7. Conclusions and Future Work

In a HWSN, human-based sensors (*i.e.*, the devices of mobile users) participate in the message routing, therefore their energy consumption becomes a critical issue. The power autonomy of these nodes (usually, smartphones) determines the time period in which they will be useful for their respective users. Trying to contribute increasing the power autonomy of these devices, this article presents a study that determines the impact that the TC message prediction has on the energy consumed by these devices, when they participate in a HWSN.

The study compared the traffic of TC messages in OLSR and OLSRp, considering the islands that are dynamically formed in a HWSN due the movements of the nodes. The simulation scenarios used in this study represent urban areas like parks, beaches, promenade, shopping malls, university campuses and theme parks, where people can use a HWSN to support interactions with other people, or between them and the environment (e.g., for detecting interest places near the people location).

The obtained results show that the use of prediction effectively helps reduce the number of TC messages and consequently the energy consumed by the nodes in all studied scenarios. In order to identify the conditions in which the predictor saves energy and the amount of these savings, we have analyzed the impact of five variables that affects the message traffic in a HSWN: node density, node speed, node mobility pattern, network degree, and shape of the working area. All of them impact on energy consumption, but at different levels of importance.

The most influencing factor was the network size, which is a combination of node density, network degree and shape of the working area. Particularly, the percentage of energy savings increases in large networks. Therefore, a high node density, a low network degree and few network partitions will tend to maximize the network size and the benefits of the TC message prediction.

The results also indicate that the motion model of the nodes affects the amount of energy consumption, and also the saved energy. Motion patterns in which nodes move in groups (e.g., in Nomadic or RPGM models) make the nodes consume less energy, and the energy saved by the predictor seems to be a percentage of energy consumed by them. Such a percentage depends on the network degree; the more the network degree, the more the percentage of energy saving.

In motion patterns where nodes move independently (e.g., in the Random Walk model), the nodes tend to consume more energy and the predictor tends to increase the percentage of saving. Motion models in which the nodes follow certain coordination in their movements (e.g., in SLAW), energy consumption and saving represents an interim point between the previous ones.

The rest of the studied variables (*i.e.*, node density, network degree, area shape, and node speed) also affect the energy consumption and savings, because they contribute to determining the network size. Summarizing, the energy consumption and the savings produced by the predictor increases with the network size and the node mobility. Using this information, designers of mobile collaborative systems can understand the expected communication capabilities of the system, and also the amount of energy that the predictor would be able to save in every work scenario. These results can also help designers to fine tune the predictor, in order to increase the percentage of saved energy in a particular work scenario; *i.e.*, to adapt the prediction process to a specific activity or place. As the predictor contributes to save energy without affecting the network throughput, software developers do not need to worry about determining when the predictor should be used. In the worst case, the use of this prediction strategy will have a neutral impact on the energy consumption of the nodes. The use of a message predictor is almost independent of the routing protocol used in the islands of a HWSN, therefore this strategy can be easily reused in several other link state routing protocols.

The use of this strategy can be combined with several other energy reduction proposals, and thus increasing energy saving. For instance, we can use the Mobile Gambler's Ruin (MGR) algorithm to predict the node mobility in a MANET (as proposed by De Rosa *et al.* [35]), and the proposed predictor to save extra TC messages. Clearly, when two or more nodes move together, which is predictable by the MGR algorithm, the proposed predictor can also avoid disseminating TC messages because it infers that the nodes are moving in a group. This strategies combination contributes to save extra energy.

The TC message predictor can also help mobile collaborative applications to save additional energy only by making an intelligent processing of the TC messages. For instance, using that information a node can identify appropriate periods to deliver messages to a destination node, and also avoid the message dissemination if the destination node is no longer part of the network. The amount of this additional saving will depend on both, the number of messages exchanged (at the application level) by the system and also on the policy that it uses to decide when delivering a message.

The next steps in this initiative consider the evaluation of this prediction strategy in real scenarios, as a way to confirm the results obtained in the reported simulations. We will then explore the use of predictors in other routing protocols for oppnets. Finally, it would be interesting to compare this proposal with others existing ones to trying identifying the best alternative to support each particular work scenario.

## Figures and Tables

**Table 1.** Simulation parameters.

**Parameter**	**Value**
Simulation time	1,800 s
Simulation areas	300 × 300 m
	30 × 3,000 m
Number of nodes	10, 20, 30 or 40
MAC Protocol	IEEE 802.11
Propagation Model	TwoRayGround
Transmission power	0.66 W
Transmission range	80 m

OLSR	UM-OLSR
Implementation	
Hello Interval	2 s
TC Interval	3 s

**Table 2.** Parameters of the mobility models.

**Parameter**	**Value**
Mobility model	Random Walk
Max. speed	1, 2, 4 or 6 mps
Max. pause	60 s

Mobility model	SLAW
Cluster ratio	25 m
Max. pause	60 s

Mobility model	RPGM
Avg. nodes per group	4
Group size deviation	2
Max. distance	15 m
Max. speed	1, 2, 4 or 6 mps
Max. pause	60 s

Mobility model	Nomadic
Avg. nodes per group	4
Group size deviation	2
Max. distance	15 m
Max. speed	1, 2, 4 or 6 mps
Max. pause	60 s
